# Natural killer cells and their exosomes in viral infections and related therapeutic approaches: where are we?

**DOI:** 10.1186/s12964-023-01266-2

**Published:** 2023-09-25

**Authors:** Mohammad Hossein Razizadeh, Alireza Zafarani, Mahsa Taghavi-Farahabadi, Hossein Khorramdelazad, Sara Minaeian, Mohammad Mahmoudi

**Affiliations:** 1 Department of Virology, School of Medicine, Iran University of Medical Sciences, Tehran, Iran; 2 Antimicrobial Resistance Research Center, Institute of Immunology and Infectious Diseases, Iran University of Medical Sciences, Tehran, Iran; 3 Department of Hematology and Blood Banking, Faculty of Allied Medicine, Iran University of Medical Sciences, Tehran, Iran; 4 Department of Immunology, School of Medicine, Tehran University of Medical Sciences, Tehran, Iran; 5 Department of Immunology, School of Medicine, Iran University of Medical Sciences, Tehran, Iran; 6 Department of Immunology, School of Medicine, Rafsanjan University of Medical Sciences, Rafsanjan, Iran; 7 Immunology Research Center, Institute of Immunology and Infectious Diseases, Iran University of Medical Sciences, Tehran, Iran

**Keywords:** NK cells, Immune evasion, Virus, Exosome, Extracellular vesicles, Innate immunity

## Abstract

Innate immunity is the first line of the host immune system to fight against infections. Natural killer cells are the innate immunity lymphocytes responsible for fighting against virus-infected and cancerous cells. They have various mechanisms to suppress viral infections. On the other hand, viruses have evolved to utilize different ways to evade NK cell-mediated responses. Viruses can balance the response by regulating the cytokine release pattern and changing the proportion of activating and inhibitory receptors on the surface of NK cells. Exosomes are a subtype of extracellular vesicles that are involved in intercellular communication. Most cell populations can release these nano-sized vesicles, and it was shown that these vesicles produce identical outcomes to the originating cell from which they are released. In recent years, the role of NK cell-derived exosomes in various diseases including viral infections has been highlighted, drawing attention to utilizing the therapeutic potential of these nanoparticles. In this article, the role of NK cells in various viral infections and the mechanisms used by viruses to evade these important immune system cells are initially examined. Subsequently, the role of NK cell exosomes in controlling various viral infections is discussed. Finally, the current position of these cells in the treatment of viral infections and the therapeutic potential of their exosomes are reviewed.

Video Abstract

**Supplementary Information:**

The online version contains supplementary material available at 10.1186/s12964-023-01266-2.

## Introduction

Viral infections cause millions of morbidities and mortalities yearly [1]. A recent estimate shows 291,243 to 645,832 influenza-associated deaths occur annually [2]. Also, viruses can increase the mortality rate in special groups of patients. For example, the hepatitis C virus (HCV) is a risk factor for cardiovascular and liver disease-related mortality in patients on maintenance dialysis [3]. Viruses are also known as significant causes of cancer. They can play their tumorigenic role via different mechanisms. Approximately 20 percent of human cancers are related to viruses, and 15 percent of cancer mortality is attributed to virus-associated cancers [4, 5]. Furthermore, dynamic balances and imbalances in the global ecosystem and relation between humans and other animals can lead to the emergence of new viral agents like the severe acute respiratory syndrome coronavirus 2 (SARS-CoV-2) and following epidemics and pandemics that highlight the importance of viral infections [6–8].

The possibility of immune system preponderance and successful viral clearance depends on effective coordination between two arms of the immune system named innate and adaptive immunity [9]. Viruses use several mechanisms to bypass or dysregulate the immune system [10]. For example, the severity of liver damage in hepatitis B virus (HBV) infection depends on the immune reaction against the infected hepatocytes [11, 12]. The other strategy of some viruses, such as human immunodeficiency virus (HIV) and Ebola virus (EBOV), is suppressing the immune system to pave the way for infection progression [13, 14].

Before adaptive immune responses are formed, the innate immune system is responsible for defending against many microorganisms [15]. Natural killer (NK) cells are lymphocytes of the innate immune system and play an essential role in the fight against cancerous and virus-infected cells [16]. Human mature NK cells were traditionally known as CD3^−^CD56^+^ lymphocytes that comprise a homogenous group of cells. However, it is well-defined that these cells undergo a differentiation process that involves functional and phenotypical changes [17]. It has been shown that patients with NK cell deficiencies are susceptible to viral infections [18]. NK cells use various mechanisms to destroy the infected cells. NK cells express multiple extracellular ligands, including the Fas ligand (FasL) and the tumor necrosis factor-related apoptosis-inducing ligand (TRAIL), to induce cytolysis of the target cell using surface death receptors [19]. Interaction of FasL and TRAIL with death receptors of virus-infected cells results in apoptosis of the target cells.

Moreover, NK cells possess granules containing perforin and granzymes that can enter the target cell and commence caspase-mediated apoptosis [16]. This case removes improper cells and helps cellular homeostasis [17]. Besides the cytotoxicity, NK cells participate in antiviral defense through releasing proinflammatory cytokines or exosomes [16, 20]. Exosomes, as a group of extracellular vesicles, can transfer cytoplasmic and membrane contents such as proteins, mRNA, and miRNA from NK cells to the virus-infected cell, exerting effects similar to those of NK cells. Therefore, they can be considered as a therapeutic target that is cell-free based.

In this review, we summarize the interactions and mutual effects of NK cells and viruses, as well as discuss the potential roles of NK cell-derived exosomes in viral infections and their potential use in antiviral therapies.

## NK cell biology

NK cells are part of the innate immune system and represent up to 20% of circulating lymphocytes in healthy humans. These cells are found in different parts of the human body, such as peripheral blood, bone marrow (BM), spleen, and non-lymphoid organs [21, 22]. NK cells are known and distinguished by CD16 (an activating Fc receptor) and CD56 (neural cell adhesion molecule (NCAM)) along with lacking a specific T cells activating receptor (TCR) and its signal-transducing adaptor, CD3ε [23]. NK cells are major histocompatibility (MHC)-independent and can kill infected or malignant cells without prior sensitization. NK cells are most similar to T cytotoxic cells and also to innate lymphoid cells (ILCs) [24].

Recent advances in NK cell biology revealed other places as a niche for NK cells, including tonsils, spleen, and (lymph nodes) LNs [25, 26]. For example, the liver is the main site for generating NK cells during fetal life from CD127^+^ CD117^+^ common lymphoid progenitors (CLP), and it has been revealed that NK cells can be generated from thymic precursors in the presence of interleukin (IL)-2 and IL-15 [27, 28]. Lin^−^ CD34^+^ CD133^+^ CD244^+^ hematopoietic stem cells (HSC) must first be differentiated into CD45RA^+^ lymphoid-primed multipotential progenitor (LMPP) to generate NK cells. LMPP differentiates into CLP by expressing CD7, CD38, CD127 (IL-7Rα), and CD10. CLPs are committed to producing Pro-B, Pre-T, natural killer cell progenitors (NKPs), and innate lymphoid cells (ILCs). To produce NK lineage, CLP expresses the IL-15 receptor β chain (CD122) as a sign of becoming NKPs, not other lineages. It has been shown that IL-15 is associated with NK cells survival, maturation, and differentiation. Transcription factors such as T-bet, Id2, Tox, Eomes, Nfil3, Ets1, PU.1, and Tcf1 are involved in NK cell maturation and development. Nfil3 and PU.1 are critical for the early stage of NK cell development. However, Eomes and T-bet are required for the later maturation stages [26, 29–32]. Through the down-regulation of CD3ε and the expression of CD7^+^ CD127^+^ NKP, they enter stage 2 of pre-NK. Based on lacking or expressing IL-1R1, stage 2 of pre-NK is divided into stages 2a and 2b, respectively. The transition to Stage 3 immature NK cells (iNK) comes with the expression of activating receptors such as NKG2D, NKp30, NKp46, and CD161. Immature NK cells develop to stage 4a CD56^bright^ NK cell, and then to stage 4b by expressing NKp80 [33–38]. CD56^bright^ NK cells are the less matured NK cell. After that, NK cells start to up-regulation of CD16 and CD94/NKG2C along with downregulation of CD56 and c-Kit (CD117) and CD94/NKG2A to become more mature (stage 6) cell (CD56^dim^ NK cell). The final stage (stage 7) of CD56^dim^ NK cells maturation is the expression of CD57 (HNK-1, Leu-7) and killer cell immunoglobulin-like receptors (KIR). CD56^bright^ NK cells are a smaller population and are mostly related to secreting inflammatory cytokine. They are predominantly found in secondary lymphoid tissues; however, CD56^dim^ NK cells constitute a significant population in the peripheral blood and are primarily associated with cytotoxic functions [30, 33, 39, 40].

## NK cell self-tolerance

Similar to other immune cells, NK cells must learn not to attack self-cells, which is known as the education process. The interaction between inhibitory Natural Killer cell receptor (iNKRs) and their ligand on self-cells leads to acquiring tolerance to self. This process helps the NK cells distinguish self-cells from infected or transformed cells, reducing their MHC class I, known as the "missing-self" hypothesis. NK cells are educated if they have an iNKR to interact with an inhibitory ligand on the self-cells [41–43]. Without inhibitory receptor-ligand interaction with self-cells, NK cells become hyporesponsive to avoid harming bodies during immune responses [41].

## NK cell receptors

NK cell receptors are mainly categorized into three subgroups. Group one is KIR which are composed of inhibitory KIRs (iKIRs) and activating KIRs (aKIRs) [44]. These highly polymorphic receptors are encoded on human chromosome 19q13.4 [45]. Inhibitory receptors are generally more important than activating receptors and play a vital role in education and missing self. KIR2DL1 (ligand HLA-C2), KIR2DL2/L3 (ligand HLA-C1), KIR2DL4 (ligand HLA-G), KIR3DL1 (ligand HLA-A and HLA-B) and KIR3DL2 (HLA-A * 03-A * 11 and HLA-F) are critical iKIR [44]. It has been shown that after influenza infection, the binding of KIR2DL1 to its ligand increased [46]. A recent study has stated a protective role of lacking KIR3DL in individuals infected with HIV and homozygous for Bw4 [47]. The well-known aKIR is KIR2DS1, which recognizes HLA-C2 as its ligand. KIR2DS1 shares high homology with KIR2DL1 [48, 49]. Although KIR2DS1 is one of the well-known activating receptors, there is a lack of evidence for its protective function in viral infection. For example, a study showed KIR2DS1-related fatal outcomes in EBOV infection [50]. However, the protective role of KIR2DS1 has been confirmed in placental human cytomegalovirus (HCMV) infection [51]. Another receptor is KIR2DS2, which recognizes HLA-C1 and has high homology with KIR2DL2/L3 [48]. This receptor protects against HCV and primary HCMV infection after kidney or bone marrow transplantation [52–54]. The adverse protective effect of KIR2DS2 has been shown in cases of Herpes simplex virus type 1 (HSV-1) infection [55]. KIR2DS3 is expressed at low levels on the NK cell surface and has no known ligand [48]. It has been shown that this receptor is related to the unsuccessful clearance of hepatitis B and C viruses [56–58]. KIR2DS4 has various ligands such as HLA-C1 and HLA-C2 alleles, HLA-A*11:02, and HLA-F [48, 59]. This receptor is associated with an increased susceptibility to HIV infection [60, 61]. KIR2DS5 is a lesser-known receptor which assumed to bind to HLA-C2 [48, 62]. It has been shown that KIR2DS5 is related to an elevated risk of symptomatic HCMV infection [63]. The last aKIR is KIR3DS1, which has been demonstrated that recognize HLA-B^Bw4^ and HLA-F as the ligand [48, 64, 65]. The protective role of KIR3DS1 in viral infections is well described. It is clear that KIR3DS1 is associated with better outcomes in HBV, HIV, and HCV infection [66–68].

The second group is Killer cell C-type lectin receptors, composed of inhibitory and activating receptors. NKG2A/CD94 is an inhibitory receptor that recognizes HLA-E as its ligand [44]. A study demonstrated that NKG2A is required to control poxvirus infection through CD8^+^ T cells [69]. It has been shown that NKG2A expression increased on the surface of NK cells by HCV and HBV infection, which can restrain immune response [70, 71]. NKG2C/CD94 is an activating C-type lectin receptor that knows HLA-E as the ligand [44]. It has been reported that NKG2C^+^ NK cells were inversely correlated with HIV viral load and exhibited anti-HIV activity [72]. Moreover, the NKG2C- NK cell frequency was higher in patients living with HIV than in HIV-exposed seronegative subjects [73]. However, a study on Brazilian individuals infected with HIV showed no association between complete deletion of NKG2C and susceptibility to HIV infection [74]. NKG2D is another activating receptor related to C-type lectin receptors. Its ligands are composed of MICA, MICB, and ULBP, which are up-regulated on the surface of infected cells [44]. It has been shown that in HCV infection, NS5A protein can stimulate monocytes through toll-like receptor (TLR) 4, which results in an increase in IL-10 production. In turn, IL-10 decreases the NKG2D expression, leading to the impairments of NK cell antiviral response [75]. HVC virus also can downregulate the expression of NKG2D ligands to avoid immune response [76]. HIV can also evade the immune response by downregulating the NKG2D ligand through its NEF protein [77].

The third comprises the natural cytotoxicity receptors (NCR), mainly categorized into NKP30, NKP44, NKP46, and NP80 [44]. NKP30 and NKP46 are expressed on NK cells before activation; however, NKP44 is expressed only after activation [44]. These activating receptors help NK cells lyse the cells expressing their ligands. The ligands for the NCRs have not been fully recognized. However, BAT-3 and B7-H6 for NKP30, PCNA, NIDOGEN-1, PDGF-DD for NKP44, and the complement factor P for NKP46 have been identified [44]. It has been shown that NKP44 and NKP46, but not NKP30, play a vital role in the lysis of influenza-infected cells through binding to hemagglutinin (HA) [78]. The previous data has shown that HCMV, via its pp65 protein, inhibits the functional activity of NKP30, resulting in NK cell inhibition [79]. NKP30 expression has been related to protection against HCV infection based on a recent study [80]. However, HCV-infected cells have been demonstrated to inhibit *ex-vivo* NK cell functions and downregulate NKP30 on the surface of NK cells [81]. NKP80 is less known than other receptors and participates in NK cell cytotoxicity by binding to its ligand, activation-induced C-type lectin (AICL) [82].

DNAX accessory molecule-1 (DNAM-1), or CD226, is an activating receptor that improves NK cell cytotoxicity by interacting with its ligands, CD155 and CD112 [83]. Lysosomal-associated membrane protein-1 (LAMP-1 or CD107) is an NK cell-related receptor that is upregulated on NK cells after cytokine secretion and NK cell-mediated lysis of target cells [84]. 2B4 and NTB-A belong to the Signaling Lymphocyte Activating Molecule (SLAM) family expressed on NK cells' surface and assist NK cell-related cytotoxicity. 2B4 ligand is CD48, and NTB-A recognizes NTB-A by homophilic interactions [85].

## NK cell in viral infections

**Fig. 1:**
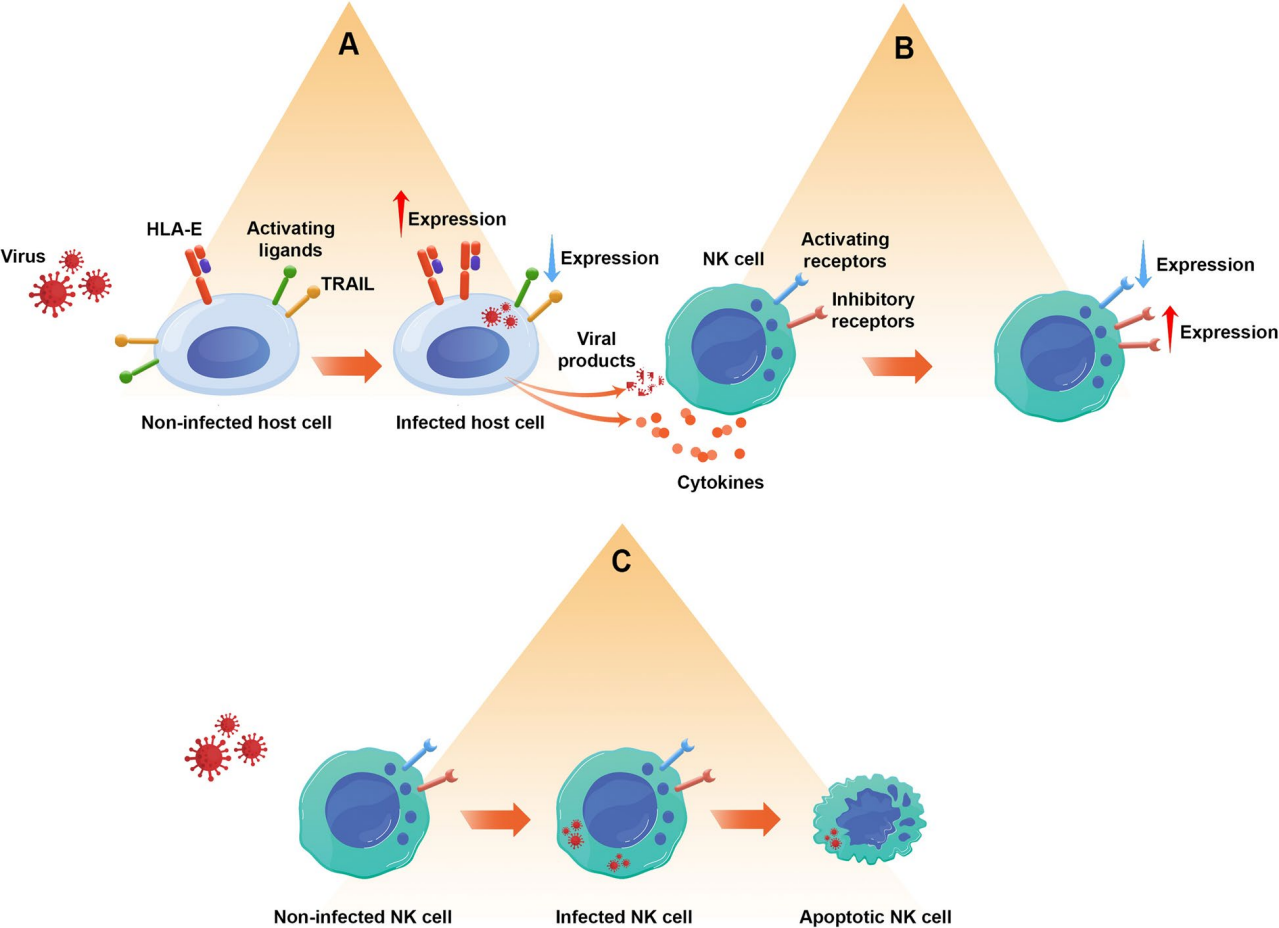
Viral infections have different ways of evading the immune response by the NK cells. **A** They can alter the proportion of ligands on the cell surface of infected cells to change the NK cell response after interacting with the infected cells from activation to inhibition. **B** Also, they can use cytokines and viral proteins to send inhibitory signals to the NK cells. **C** Some viruses can also directly infect the NK cells and induce apoptosis

NK cells are one of the first barriers against viral infections. Their different methods to detect and annihilate the infected cells have been discussed previously. Conversely, viruses have evolved to develop different mechanisms to evade the NK cell response (summary in Fig. 1 and with more details in Table 1). This section describes the interaction between different viral families and NK cells.

**Table 1:** Viral proteins and their role in evading NK cell's response

Virus	Protein	Mechanism
**B19**	?	Counteracts NK cell differentiation, activation, and cytotoxicity by decreasing the expression of TNF-α mRNA [86]
**BK**	?	Decreases KIR3DS1 activating receptor [87]
**HPV**	E6 and E7	Decrease the production of IFNs [88–90] Inhibits inflammasome-induced inflammation [90, 91]
	?	Downregulates the activating receptors NKp30, NKp46, and NKG2D [92]
	?	Weakens the cytotoxic capacity of NK cells [93]
**HCMV**	UL16	Avoids being detected by NK cells by binding to ULBP1 and MICB [94]
	UL18	binds to an MHC-I inhibitory receptor LIR-1 [95]
	UL40	upregulates the expression of the HLA-E [96]
	UL141	Downregulates the surface CD112 and CD155 [97, 98] Downregulates surface TRAIL death receptors [99]
	UL142	downregulates the cell surface amount of MICA by retention in the cis-Golgi apparatus [100]
	UL148A	Downregulates the MICA by lysosomal degradation [101]
	US18 and US20	Lysosomal degradation of MICA and B7-H6 [102, 103]
	US9	Downregulates MICA^*^008 by proteasomal degradation [104]
	US12	Targets different NK cell ligands, adhesion molecules, and cytokine receptors [105]
	pp65	Weakens the cytotoxic capacity of NK cells by disrupting the CD3ζ signaling pathway [79]
	gp34, gp95, gpRL13, and gp68	Avoids ADCC by acting as CD16 (FcγIII receptor) analogs [105]
**HSV and VZV**	?	Decreases MICA, ULBP1, ULBP2, and ULBP3 [105]
	gD	Avoids being detected by NK cells by decreasing CD112 [106]
	gE-gI complex	Avoids ADCC by acting as CD16 (FcγIII receptor) analogs [105]
**HHV-7**	U21	Downregulates the surface levels of MICA and MICB [107] Reduces the ULBP1 protein by lysosomal degradation [107]
**KSHV**	ORF54-encoded protein	downregulates an unknown ligand of NKp44 [108]
	vMIP-II (vCCL2)	acts as an antagonist for Fractalkine and RANTES chemokine receptors to inhibit NK cell migration [109]
	K5	Downregulates MICA and AICL cell surface levels by intracellular retention [110]
**SARS-CoV-2**	Spike	Increases the surface expression of HLA [111]
**DENV**	?	Avoids being detected by NK cells by upregulating MHC-I [112]
**HCV**	?	downregulates the expression of NKG2D and NKp30 [113]
**RSV**	?	downregulates the expression of NKG2D and NKp44 [114]
**EBOV**	VP24 and VP35	Act as antagonists to IFNs [115, 116]
**Marburg virus**	VP40	Blocks the type I IFNs signaling [117]
**HIV**	nef	Decreases the cell surface amount of MICA, ULBP1, and ULBP2 [77] Decreases the NK cell activity by intracellular retention of NKp44 ligands [118]
	nef and vpn	downregulates the expression of NTB-A and CD155 [119–121]
	?	Impairs the expression and function of NKG2D [122]

### Parvoviridae

Members of the *Parvoviridae* family are non-enveloped viruses with a linear single-stranded DNA genome that can infect a diverse array of animals, from arthropods to vertebrates [123]. B19 is the most well-known member of this family because of its involvement in various diseases such as hydrops fetalis, aplastic anemia, erythema infectiosum, myocarditis, and rheumatologic diseases [124–126]. Another member of this family is the Human bocavirus (HBoV), which is assumed to contribute to respiratory and gastrointestinal complications [127].

Alteration of host cytokine status after B19 infection affects the NK cells. Infection of monocytes by B19 results in decreased expression of tumor necrosis factor (TNF)-α mRNA [86]. TNF-α enhances IL-2 effects to cause NK cell differentiation, activation, and cytotoxicity [128]. Infection of human airway epithelial cells by HBoV-1 increases the release of IL-18 [129]. IL-18 promotes NK cell proliferation [130] and provokes antiviral activity of NK cells by inducing the production of IFN-γ [131]. Additionally, Parvovirus infection may affect the number of NK cells. A comparison of B19-infected renal transplant recipients with non-infected recipients indicated a considerably lower number of lymphocytes, including NK cells [132]. Moreover, a significant decrease in the number of NK cells, as well as T and B lymphocytes, was observed in a patient with B19-related hepatitis negative for hepatitis A, B, and C viruses [133].

### Polyomaviridae

The *Polyomaviridae* family is a group of non-enveloped viruses with a circular double-stranded DNA genome that is only 5.5 kbp in size. The capsid is icosahedrally shaped and is constructed of 72 pentameric capsomers. The *Polyomaviridae* family consists of 5 genera and 37 species, which are infectious to avian and mammalian species [134]. Their genome encompasses three parts: (I) The regulatory region contains promoters and the origin of replication that regulates the expression of the other parts. (II) The early region encodes early proteins, including large and small T antigens. (III) The late region is expressed from the complementary region and encodes capsid and agnoprotein [135]. To date, 14 human polyomaviruses (HPyVs) have been identified. Some of them are known as human pathogens [134]. For instance, the BK virus is involved in nephritis pathogenesis, JC virus in progressive multifocal leukoencephalopathy, MCPyV in Merkel cell carcinoma, and TSPyV in Trichodysplasia spinulosa. HPyVs 6 and 7 are also suggested to be potential contributors in pruritic rashes [136].

A correlation was observed between the genotype of activating NK cell immunoglobulin-like receptors and controlling BK virus infection in renal transplant recipients. Upregulation of HLA-F expression has led to enhanced binding of KIR3DS1 to BK virus-infected cells and, thus, activation of KIR3DS1^+^ cells [137]. In patients with BK virus-associated nephritis, NK cells possessed lower KIR3DS1 activating receptors than a control group of recipients negative for BK virus infection. Researchers also found that the telomeric part of KIR haplotypes is associated with infection, suggesting an NK cell genetic predisposition to BKV infection [87]. Solenocytes of Polyomavirus-infected mice with severe combined immunodeficiency (SCID) showed many MHC-1 molecules that can mitigate NK cell activity. These mice finally developed an NK cell-resistant lymphoproliferative disease [138]. Infection of NK cell-deficient mice resulted in faster tumor development than TCRβ × δ KO mice. The established cell line from the developed expressed Rae-1, a cellular stress molecule that acts as an NKG2D ligand, resulting in the efficient destruction of these cells by NK cells [139].

### Papillomaviridae

The *Papillomaviridae* is a family of dsDNA viruses with a non-enveloped icosahedral virion. They are classified based on the genetic similarity of the major capsid protein (L1) [140]. Human Papillomaviruses (HPVs) are well-known for their ability to cause different malignancies in humans, especially anogenital cancers. For example, almost all cervical cancers are HPV + [141]. The main transmission route is through sexual activity, but the viruses can spread via skin-skin contact. Up to now, more than 200 HPV types have been detected, of which at least 14 high-risk genotypes have been recognized. Interestingly, about 80% of people contract the virus in their lifetime [142].

Despite the efforts of NK cells to recognize and kill the cancerous cells, HPV can modulate that. For instance, a comparison of the NK cell count in tissues of women with cervical cancer showed that patients with higher HPV viral load had lower numbers of NK cells while patients with lower viral load had higher numbers of NK cells [143]. HPV oncoproteins E6 and E7 can downregulate the transcription of type 1 IFNs. E6 is also shown to downregulate the expression of IFN-κ, a member of type 1 IFNs [88]. They can also suppress the IL-18-induced IFN-γ by binding to the IL-18 receptor [89]. Viral oncoproteins also can inhibit inflammasome activation to avoid NK cell activity by cytokines. E6 degrades the p53 to inhibit the transcription of Interferon Regulatory Factor 6 (IRF6) and thereby decreasing the IL-1β [90]. E7 interaction with Interferon Gamma Inducible Protein 16 (IFI16) and TRIM21 and recruitment of the E3 ligase TRIM21 to degrade the IFI16 inflammasome by ubiquitination leads to reduced IL-18 [91]. A remarkable downregulation of NK cell activating receptors NKp30, NKp46, and NKG2D in the high-grade squamous intraepithelial lesion and cervical cancer was observed [92], and NK cells in HPV infected cervical cancer microenvironment show inadequate cytotoxicity [93]. Moreover, HPV seems to induce carcinogenesis by stimulating inflammatory reactions by increasing KIR2DS5 activating receptor that is shown to be associated with cancers [144].

### Herpesviridae

The family *Herpesviridae* is an extensive group of double-stranded DNA, enveloped viruses able to establish persistent infection in the immune competent hosts. This family is further classified into three main branches according to genome organization, biological characteristics, and cellular tropism: Alpha-, Beta-, and Gammaherpesviruses [145]. HSV1 and HSV2 are the most well-known members of this family, known for their role in various complications such as cold sores, herpetic keratitis, and encephalitis. The other member, which infects more than 90% of the world population, is the Epstein-Barr virus (EBV) [146], a lymphotropic virus that causes infectious mononucleosis. EBV was first discovered in cultured African endemic Burkitt’s lymphoma cells [147]. To date, its association with different types of cancer, such as nasopharyngeal and gastric cancer, has been found [148]. HCMV is the other important and prevalent herpesvirus that poses a dangerous threat to immunocompromised individuals by causing various clinical manifestations, including retinitis, gastroenteritis, hepatitis, pneumonitis, transplant rejection, atherosclerosis and damaging fetus [149].

In 1989, Biron et al*.* reported a 13-years-old girl with NK cell deficiency that had severe infection with HSV, Varicella Zoster virus (VZV), and HCMV [150]. Another study on children with herpetic encephalitis found that all patients had NK deficiencies [151]. Mutations in GATA-2, which is required for NK cell maturation [152], hinder early differentiation of CD56^bright^ NK cells, which predisposes individuals to herpesvirus infections and mutations in minichromosome maintenance complex component 4 (MCM4) or IRF8 inhibits the maturation of CD56^dim^ NK cells, which makes individuals prone to EBV-associated lymphoproliferative diseases [153]. Herpesviruses developed several mechanisms to evade NK cells. It has been observed that patients with NK cell deficiencies are prone to life-threatening infections by different Herpesviruses. MICA, MICB, and ULBP1 are upregulated in HCMV-infected cells. Placement of these proteins on the surface of infected cells triggers NK cells via binding to NKG2D [105]. UL16 can bind to ULBP1 and MICB to neutralize their effects, but not MICA due to extracellular α2 domain differences [94]. The other HCMV glycoprotein UL142 is able to inhibit MICA and ULBP3 [105]. UL148A has recently been found to downregulate MICA through lysosomal degradation. However, it cannot solely act, and an unknown viral factor seems essential for its proper function [101]. US18 and US20 are other HCMV-coded proteins that target MICA and the NKp30 ligand B7-H6, which induces MICA lysosomal degradation [102, 105]. HCMV US9 can selectively target MICA^*^008 to its proteasomal degradation [104]. A new family of HCMV genes called the US12 gene family was introduced recently that targets NK cell ligands, adhesion molecules, and cytokine receptors [105]. HSV-1 infection decreases the cell surface levels of MICA, ULBP1, ULBP2, and ULBP3 in a non-elucidated process [105]. The expression of MICA and MICB is decreased in HHV-7-infected astrocytoma cells. ULBP1 was transferred by HHV-7 to a lysosomal compartment, which resulted in its degradation. [107]. DNAM-1 ligands are the other targets of Herpesviruses. UL141 is reported to downregulate cell surface expression of DNAM-1 ligands CD112 and CD155 [105]. It has been observed that infection with laboratory HCMV strains AD169 and Towne that faced deletion mutations in the UL141 gene results in higher sensitivity to NK cells compared with infection with clinically isolated strains [154]. Moreover, UL141 downregulates surface TRAIL death receptors required for TRAIL-induced NK cell-mediated apoptosis [99]. In the case of Alphaherpesviruses, gD stimulates the degradation of CD112, and strains lacking gD failed to evade recognition by DNAM-1 [155]. Interaction of the HCMV major structural protein pp65 with NKp30 disrupts the CD3ζ signaling pathway and enfeebles the NK cell cytotoxicity [79]. Also, It has been seen that the ORF54-encoded protein of Kaposi sarcoma herpesvirus (KSHV) downregulates an unknown ligand of NKp44 [108]. Herpesviruses encode CD16 (FcγIII receptor) analogs to impede the antibody-dependent cellular cytotoxicity (ADCC). These proteins are the gE-gI complex of HSV and VZV, gp34, gp95, gpRL13, and gp68 of HCMV [105]. EBV BCRF1 encodes IL-10 to hamper NK cell activation [156]. KSHV inhibits NK cell migration by encoding a viral chemokine, vMIP-II (vCCL2), which acts as an antagonist for chemokine receptors of Fractalkine and RANTES [105, 109]. The other KSV protein, K5, retains the NKG2D and NKp80 ligands MICA and AICL within the cell to avoid their presence on the cell surface [110].

To combat MHC-I-dependent activity, herpesviruses developed various strategies. For instance, the HCMV UL18 protein binds to leukocyte immunoglobulin-like receptor 1 (LIR-1), an MHC-I inhibitory receptor. Furthermore, HCMV UL40 upregulates the expression of the nonclassical MHC-I molecule HLA-E to protect the infected cell from the activation of CD94/NKG2A^+^ NK cells [105]. Polymorphism in the UL40 sequence affects the HLA-E/NKG2 binding affinity since the UL40 protein with polymorphism showed less affinity to NKG2 receptors [157].

### Coronaviridae

The *Coronaviridae* family consists of enveloped viruses characterized by single-stranded positive-sense RNA, capable of infecting both humans and animals [158]. This family is divided into four subfamilies, alpha (α), beta (β), gamma (γ), and delta (δ) coronaviruses (CoVs). Seven members of this family are known to infect humans: NL63 and 229E in the alpha-CoV and HKU1, OC43, SARS-CoV-1, Middle East respiratory syndrome, (MERS-CoV), and SARS-CoV-2 in the beta CoV. Thus far, three pandemics have occurred among the members of this family, SARS-CoV-1, MERS-CoV, and SARS-CoV-2; the latter is more infectious and contagious and is responsible for the latest pandemic, named Coronavirus disease (COVID-19), leading to the death of millions of people worldwide [159].

Patients infected with SARS-CoV-2 had low numbers of CD56^dim^ and CD56^bright^ NK cells that might result from trafficking to the lungs via CXCR3, CXCR6, and CCR5 chemokines [160, 161]. NK cells in the lung are active and proliferative, especially the CD56^bright^ subset, and thus, they appear to be cytokine-derived since CD56^bright^ cells respond more vigorously to cytokines [160]. The activation of these cells appears to be triggered by IL-6, IL-6R, and IL-18 [162]. Moreover, prolonged activation of NK cells can contribute to their dysfunction. It has been suggested that prolonged stimulation of NK cells by IL-15 may cause NK cell dysfunction, probably by epigenetic reprogramming. In addition, surface expression of HLA-E is elevated in patients infected with SARS-CoV-2 [160]. This is attributed to the Spike 1 (S1) protein [111]. On the other hand, viral Non-structural protein 13 (Nsp13) encodes an HLA-presented peptide that hampers the inhibition of NKG2A^+^ NK cells and, thus, helps the NK cells to restrain the infection [163]. Antibodies against SARS-CoV-2 trigger ADCC of infected cells by NK cells, and this was even higher in infected patients than in those who received an S1 coding vaccine. Cross-reactive antibodies in sera collected before the pandemic also could stimulate ADCC [164].

The immunophenotype of NK cells might affect the severity of SARS-CoV-2 infection. Maruthamuthu et al*.* have linked the absence of KIR3DL1^+^HLA-Bw4^+^ and KIR3DL2^+^HLA-A3/11^+^ and the plethora of KIR2DS1^+^KIR2DS5^+^ to the severe infection [165]. Another study reported more KIR2DS4 in severe and KIR3DS1^+^HLA-B^*^15:01^+^ in mild patients compared with controls [166]. Furthermore, KIR2DS2 and HLA-C1 complex exhibited potent effects against COVID-19 adverse effects [167]. A study of hospitalized and outpatients has suggested that NKG2C is a pivotal actor in limiting the severity of COVID-19 since KLRC2^del^ is observed more in hospitalized patients. It also demonstrated that the HLA-E*0101 variant is more prevalent in hospitalized patients [168].

Intriguingly, a high probability of HCMV reactivity in SARS-CoV-2 infected patients was observed. Although the mechanism has not been completely discovered yet, it is suggested that the high proportion of NKG2C^−^/NKG2A^+^ cells in patients with COVID-19 is associated with higher HCMV viremia and mortality [169, 170].

### Flaviviridae

The *Flaviviridae* family consists of enveloped single-stranded RNA viruses that are further classified into four genera: Flavivirus, Hepacivirus, Pegivirus, and Pestivirus. Flaviviruses such as Zika virus (ZIKV), West Nile virus (WNV), Dengue virus (DENV), Yellow fever virus (YFV), Japanese encephalitis virus (JEV), Tick-borne encephalitis virus (TBEV) are mosquito-borne and tick-borne viruses that mostly infect humans and cause a wide range of symptoms from mild febrile condition to severe hemorrhagic fever. The only human-infecting member of the Hepacivirus is HCV. Members of the *Flaviviridae* family are ubiquitous and annually cause millions of new cases [171].

NK cell response to Flavivirus is restricted to less differentiated CD56^bright^ and CD56^dim^ cells since they are more sensitive to IL-12, IL-15, IL-18, and type I IFNs [17]. Utilizing West Nile virus (WNV) like particles and WNV-infected cells indicated that the interaction of Flavivirus envelope (E) protein with NKp44 receptor leads to IFN-γ production and activation of NK cells [172]. The addition of Anti-DENV monoclonal antibodies to DENV-infected Raji cells with reduced MHC-I expression in the presence of PBMCs taken from a patient infected with DENV showed increased cytotoxicity, while the addition of sera from healthy individuals has not shown such result [173]. Similar the SARS-CoV-2, the immunophenotype can also affect NK cell response to Flaviviruses. It has been shown that the inhibitory receptor KIR2DL3 and homozygosity of its ligand HLA-C1 are associated with HCV clearance [17, 174]. While NKG2A^+^ and CD94^+^ cells were elevated in acute HCV infection, they were not involved in the anti-viral response. Conversely, NKp30^+^, NKp46^+^, CD161^+^, and NKG2D^+^ were detected in patients that successfully resolved infection [175]. Another study also showed NKp56^high^ NK cells had a higher cytotoxicity IFN-γ secretion ability than NKp56^dim^ cells [176].

DENV can evade NK cell response by upregulating MHC-I through an unknown mechanism, but the viral non-structural proteins are proposed to be involved in this process. Besides reducing cytotoxicity, aggregation of MHC-I may increase affinity to NK cell inhibitory receptors [112]. Infecting IL-12p40^−/−^ and IL-18^−/−^ mice with DENV decreased IFN-γ release and escalated the disease severity [177]. DENV-infected cells expressed fewer NKp30 and NKp46 ligands to avoid NK cell activation [178]. In the case of HCV, it has been observed that high viral load was associated with NK cell exhaustion through elevating exhaustion marker programmed cell death protein (PD)-1 expression [179]. In addition, cell-to-cell contact between HCV-infected human hepatoma cell line and NK cells reduces degranulating and IFN-γ production ability and downregulates expression of activating receptors such as NKG2D and NKp30 [113].

### Orthomyxoviridae

Viruses in the *Orthomyxoviridae* family have enveloped viral particles that harbor a negative-sense, single-stranded, and segmented RNA genome. This family is classified into seven genera: influenza virus (IV) A, B, and C; Thogotovirus; Isavirus; and a new genus Quaranjavirus. IVs are responsible for numerous endemics and pandemics in history with tremendous mortality [180]. Symptoms can be different from a cold-like disease to lethal pneumonia. These viruses are highly prone to genetic alterations and can evade preexisting immunity in the host’s body [181].

IV uses HA glycoprotein to enter the host cell by attaching to α-2,3 and/or α-2,6 linked terminal sialic acids. IV can infect the NK cells since both sialic acids are present at the surface of NK cells. Also, the activation NKp46 receptor possesses α-2,6 terminal sialic acids. While NK cells can detect IV-infected cells through the attachment of NKp46 to HA and act against them, IV can counterattack the NK cells by infecting them in this way [16]. Mice lacking the NCR1 (NKp46 in humans) gene died after infection by IV A H1N1 while NK cells were accumulated at the site of infection while NCR1^+^ mice survived, showing the pivotal role of NKp56 in the eradication of IV infection [182]. Interaction of NK cells with intact or free HA leads to the downregulation of NKp30 and NKp46 to mitigate cytotoxicity.

Furthermore, IV induces apoptosis of NK cells that can be prevented by caspase-3 inhibitor (Z-DEVD-FMK) [183], which is supported by evidence noting depletion of peripheral NK cells in IV-infected patients [16]. IV also affects the cytokine and chemokine status of NK cells. It has been shown that IFN-γ, GM-CSF, MIP-1α, MIP-1β, and RANTES are downregulated in IV-infected NK cells [184]. However, data are debating about IFN-γ and TNF-α since their upregulation in the presence of IL-15 is reported [16].

Gene expression and NK cell phenotype affect the anti-influenza response. A cohort study on the expression profile of the KLRD1 gene showed that this gene is downregulated in symptomatic IV-shedders and depicted a negative association with the severity of symptoms. Thus, the expression profile of this gene can be a predictor of influenza severity [185]. CD56^bright^CD49a^+^ NK cells exhibited a more efficient response than CD56^bright^CD49a^−^ against IV infections, as assessed by CD107a expression analysis [186]. Interestingly, the lethal outcome of infecting TLR7-KO mice with IV showed that capacity of NK cells for activation, interferon (IFN)-γ production, and cytotoxicity is related to TLR-7 [187]. Therefore, we suggest the evaluation of TLR-7 variants and mutations to understand their impact in the severity of influenza in patients.

### Pneumoviridae

Members of the *Pneumoviridae* family are a group of large enveloped negative-sense RNA viruses that were previously a subfamily within the *Paramyxoviridae* family. After reclassification in 2016, they have become an independent family with two genera Orthopneumovirus and Metapneumovirus. These viruses mainly infect mammals, but some can also infect birds [188]. Human respiratory syncytial virus (RSV) is a human pathogen in this family that is notorious for causing the most frequent viral acute lower respiratory tract infections in infants with a high burden of hospitalization, pediatric intensive care unit (PICU) admission, and mortality worldwide [189].

In an in vitro study, RSV infected about 90% of NK cells, showing its ability to infect various subsets of NK cells [190]. In contrast to other immune cells, RSV-infected NK cells do not release viral particles [16]. However, NK cell phenotype can be indirectly affected by RSV. Pre-incubation of NK cells with RSV resulted in the downregulation of NKG2D and NKp44 [16, 114]. RSV infection also upregulates MICA on the lung epithelial cells. This might be due to the increased secretion of IFN-γ that affects MICA expression with a negative impact mechanism that eventually inhibits NK cells [191]. RSV-infected infants expressed LILRB1 higher than controls. It may implicate a high risk of severe RSV infection. RSV infection of lung epithelial cells upregulates the LLT1 gene that stimulates CD161 [114, 192].

Expression of CXCL10 on lung epithelial cells leads to infiltration of NK cells to the lungs and, consequently, reduces the number of NK cells in the peripheral blood [114]. When co-culturing with RSV-infected cells, neonatal NK cells showed decreased perforin secretion compared with adults. However, CD107 expression is upregulated in neonatal and adult RSV-infected NK cells [16]. IFN-γ production negatively impacts antibody production in RSV-infected infants [193]. Blocking IFN-γ production and depletion of NK cells, T cells, or both exhibited a remarkable enhancement of anti-RSV antibody production [114]. It shows the role of IFN-γ secretion by NK and T cells in APC inhibition [114]. Moreover, IFN-γ increased IL-15 expression in epithelial cells that improved CD8^+^ T cells. In conclusion, IFN-γ secreting NK cells enhance the antigen-specific CD8^+^ T cells that eventually inhibit NK cell activity [114, 194].

### Filoviridae

Viruses in the *Filoviridae* family are non-segmented negative-stranded famous for causing fatal hemorrhagic fever outbreaks of zoonotic origin [195]. To date, a total of 12 members have been identified within this family. The medically important members are included within the two groups named EBOV and Marburg virus (MARV) [196]. EBOV includes Bundibugyo ebolavirus, Reston ebolavirus, Sudan ebolavirus, Taï Forest ebolavirus, and Zaire Ebolavirus. Only Bundibugyo, Sudan, and Zaire EBOVs were attributed to human infections, with a case fatality rate of 25%, 50%, and 80%, respectively [197]. Filovirus outbreaks have led to the loss of tens of thousands of lives [198].

Host cells infected with the EBOV are reportedly resistant to being destroyed by NK cells [199]. EBOV infection suppresses the release of type I IFNs and IL-12 [200]. The VP24 and VP35 of EBOV disrupt NK cells' maturation and activation [115, 116]. However, EBOV viral-like particles (VLPs) cannot cause the same result, indicating the prominent role of cytokines in NK cell-mediated immunity to EBOV [200]. NK cells use NKp30 and probably NKG2D to recognize EBOV VLPs. In contrast, EBOV downregulates ligands of these receptors to avoid NK cell activation [201]. To counteract NK cell activation via type I IFNs signaling, Marburg virus VP40 hinders the activation and function of Janus kinase 1 (JAK1), which results in the impaired type I IFN-induced phosphorylation of STAT1 and STAT2 [117].

One of the main characteristics of Ebola disease is severe immunosuppression due to lymphocyte apoptosis. NK cells are shown to infiltrate EBOV-infected mice organs and kill infiltrating T cells [202]. Moreover, elevated levels of IFN-γ were observed in patients with hemorrhagic shock in contrast to survivors [200]. In this case, the similarity of mRNA levels of IFN-γ in both groups suggests that the difference in the translation level may contribute to the disease fate [200, 203]. EBOV glycoprotein VP40 triggers NK cells through an IL-12 and IL-18-dependent manner, leading to a higher increase of TNF-α, IFN-γ, perforin, and granzyme B by CD3^−^CD56^+^ cells [204].

### Retroviridae

Retroviruses are enveloped viruses containing a dimer of single-stranded positive-sense RNA and a reverse transcriptase enzyme. They have a restricted host range and among them, human T-lymphotropic viruses (HTLV), HIV, and simian foamy viruses (SFV) are able to infect humans. According to the epidemiological, phylogenetic, and genomic characteristics investigations, it is assumed that HIV originated from the simian immunodeficiency virus (SIV) [205]. HIV belongs to the genus Lentivirus; it is estimated that 37.9 million people live with HIV infection [206]. HIV is classified into HIV-1 and HIV-2, originating independently from chimpanzee simian immunodeficiency virus (SIV) and Old-World monkey SIV, respectively. HIV poses a considerable threat to the body since it cripples the immune system by triggering pyroptosis through caspase-1 activation in lymphoid CD4 T cells [205].

HIV shows tropism to CD4^+^ T cells and other immune cells such as dendritic cells and macrophages. HIV has developed various mechanisms to avoid NK cell-induced immune response. NK cells release CCR5 ligands, including β-chemokines, CCL3, CCL4, and CCL5, that hinder the attachment of the virus to the cells [207]. The phenotype variation of NK cells is related to different innate immune conditions in HIV-infected patients. For example, combining HLA-B Bw4-80I KIR3DL1*h or KIR3DS1 improves immune response. In the case of KIR3DS1^+^ NK cells, an HLA-B Bw4-80I-dependent suppression of viral replication has been observed, showing the important role of NK cells in anti-HIV immunity [17]. Characterizing the KIR and HLA genes DRB1*11 and DRB1*12 has shown that the inhibitory KIR2DL5B and the HLA-DRB1*12 allele may cause protection against HIV-1 in seronegative couples. Also, KIR gene haplotype (AA and Bx) is not associated with infection in serodiscordant partners [208]. While MHC-I downregulation happens in HIV infection, HLA-C and HLA-E85 are exempted from downregulation, resulting in impaired recognition of the infected cells [209]. Furthermore, although NKG2D ligands are upregulated in HIV-infected cells, viral accessory proteins hamper the activation of NK cells [17, 210]. HIV nef protein counteracts the recognition by NK cells via decreasing the cell surface amount of MICA, ULBP1, and ULBP2 [77]. Infection of CD4^+^ T cells with HIV has resulted in the downregulation of NKp44 ligands via nef-mediated intracellular retention of these ligands [118]. Nef also, alongside vpu, downregulates the expression of NTB-A and PVR (CD155) [119, 120]. HIV-infected cells release soluble ligands for the NKG2D receptor that downregulates and functionally impair NKG2D receptors. Patients receiving highly active antiretroviral therapy have shown improved NKG2D function and lower plasma levels of the soluble ligands for the NKG2D [122].

HIV infection can alter the NK cell receptor functions. For instance, a remarkable loss of CCR7^+^CD56^bright^ NK cells that results in higher viral load has been shown in treatment-naïve patients [211]. In addition, the loss of CD56^dim^ cells has also been reported [211]. It has been shown that treating HIV-infected patients changes the CD56^bright^ and CD56^dim^ population in the body [212]. The SIV infection in the animal models caused the consolidation of CD56^−^CD16^+^ NK cells in lymph nodes [17, 213].

## The crosstalk between NK cells and virus-infected cells via exosomes

### Exosomes

The cells interact with each other in various ways, such as cell–cell contact, secretion of soluble molecules, and by secreting exosomes. Exosomes are a subtype of extracellular vesicles involved in intercellular communication [214]. Most cell populations can secrete these Nano-sized vesicles, and it was demonstrated that they exert the same effects as the cell from which they are secreted [215]. Exosomes have lipid bilayer structures and can carry various macromolecules such as miRNAs, mRNAs, proteins, etc. In addition, these vesicles can carry some molecules of the origin cell on their surface [216].

### The properties of NK cell-derived exosomes

**Fig. 2:**
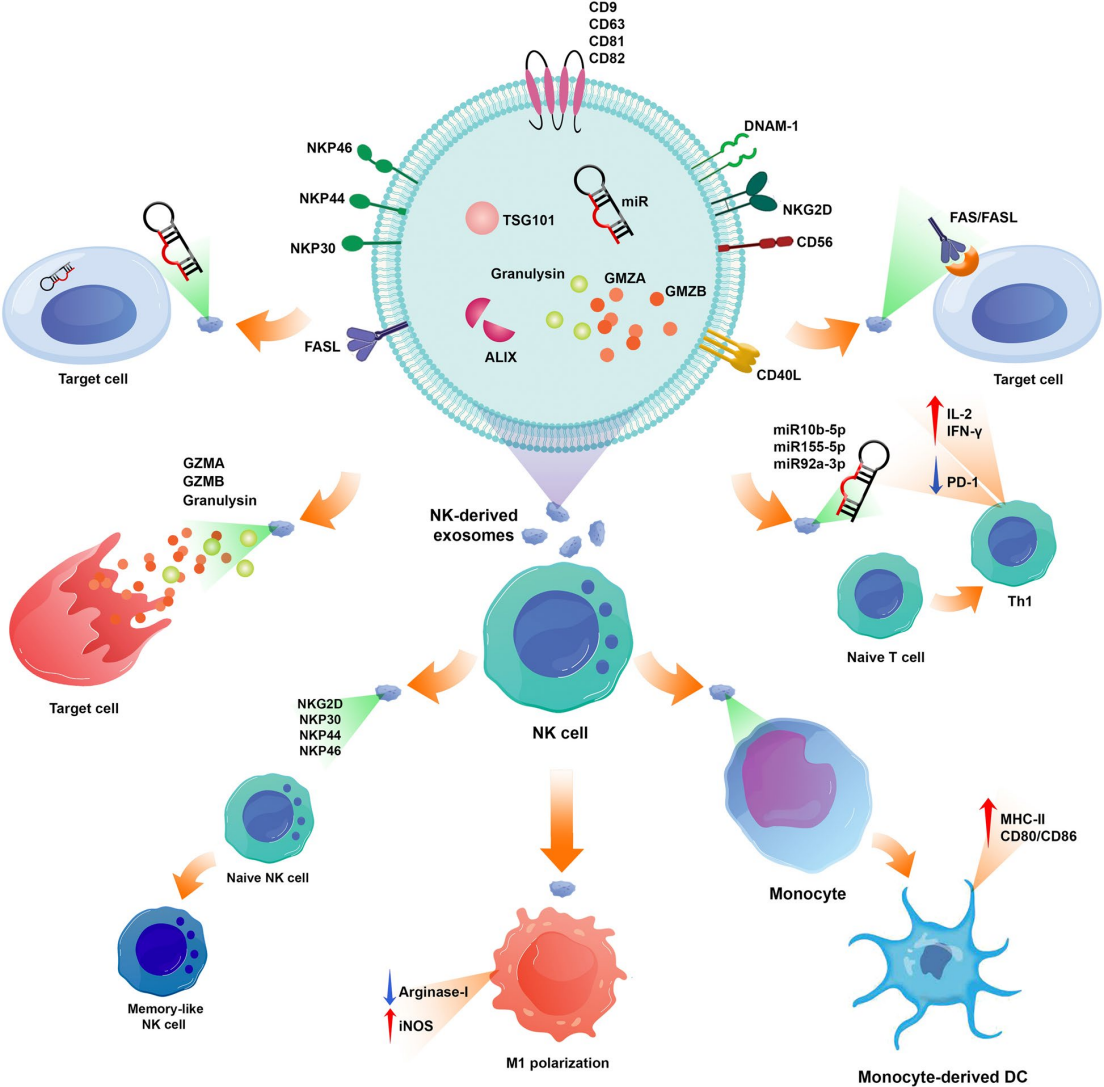
NK cells can secrete exosomes with various cargo. These exosomes contain TSG101, Alix, CD9, CD63, CD81, and CD82. In addition to typical exosome markers, NK cell-derived exosomes exhibit specific biomarkers such as CD56, DNAM-1, NKp30, NKp44, NKp46, and NKG2D. NK cell-derived exosomes contain perforin and express FasL, which contribute to the cytotoxicity effects of these vesicles. Cytotoxic proteins such as perforin, granzyme A, granzyme B, and granulysin can induce apoptosis in target cells. Since the mechanism of action of NK cells and their exosomes against tumor and virus-infected cells is relatively similar, it seems that NK cell exosomes use the following mechanisms to fight against virus-infected cells. NK cell derived-exosomes can overexpress the NK activating receptors NKp30, NKp44, NKp46, and NKG2D and be able to educate naïve NK cells, which evolve into a memory-like state, with increased cytotoxicity and enhanced tumor-killing capacity. NK cell-derived exosomes were rich in miR-10b-5p, miR-155-5p, or miR-92a-3p. T cell response can be the putative target of these small RNAs. So, NK cell-derived exosomes promote Th1 differentiation and increase IL-2 and IFN-γ production. On the other hand, the polarization of monocyte to monocyte-derived dendritic cells can be affected by NK cell-derived exosomes, and these vesicles increased the expression of MHC-II and CD86 on these cells. NK cell-derived exosomes can shift macrophage polarization to M1. As mentioned, the existence of FasL and TRAIL on the surface of NK cell-derived exosomes and cargo of perforin, granzyme A, granzyme B, and granulysin were confirmed by multiple studies supporting direct cytotoxic effects of NK cell exosomes in the face of target cells. miRNAs are another tools that these valuable exosomes use to inhibit target cells

Like other immune cells, NK cells can also secrete exosomes with various cargo and affect the target cells (Fig. 2). Exosomes from different sources have some common characteristics; for example, they contain multivesicular bodies associated proteins such as TSG101 and Alix or some tetraspanin glycoproteins like CD9, CD63, CD81, and CD82. Although definitive biomarkers for NK cell-derived exosomes have not been identified yet, some studies demonstrated that in addition to typical exosome markers, NK cell-derived exosomes exhibit some specific biomarkers such as CD56, DNAM-1, NKp30, NKp44, NKp46, and NKG2D [44].

As mentioned, exosomes exert the effects of the cell they originated, so NK cell-derived exosomes are expected to have cytotoxic effects on target cells. Lugini et al*.* isolated exosomes from resting and activated NK cells and showed that these exosomes contain perforin and express FasL, which contribute to the cytotoxicity effects of these vesicles [217]. Jong et al*.* isolated a large quantity of NK cell-derived exosomes and assessed their cytotoxic effects. They demonstrated that NK cell-derived exosomes contain several cytotoxic proteins such as perforin, granzyme A, granzyme B, and granulysin. These cytotoxic proteins can activate the caspase-dependent pathway in target cells and induce apoptosis [218]. Another study introduced other effector factors for NK cell-derived exosomes' cytotoxic functions, such as TRAIL, fibrinogen, and β-actin. Moreover, the proteome analysis in this study confirmed that these exosomes contain NKG2D and FasL, and these molecules can cause cytotoxic effects on target cells [219]. Other studies indicated that NK cell-derived exosomes can carry IFN-γ and TNF-α [220, 221]. Mass spectrometry and cytokine analysis performed by Federici et al*.* demonstrated that NK cell-derived exosomes express markers that contribute to their cytotoxic effects, trafficking, adhesion, and activation of immune responses. These markers contain NKG2D, CD94, perforin, granzymes and CD40L [222].

### Mechanism of action of NK cell-derived exosomes

As mentioned, NK cells have roles in defense against tumors and viral infections. Due to the availability of different cancer cell lines, as well as the fact that cancer is non-contagious and safer than viral infection, most of the studies related to NK cells have focused on their anti-tumor properties. So, the effects of NK cell-derived exosomes were assessed on different cancer cells. Many studies investigated the cytotoxic effects of NK cell-derived exosomes on both hematologic and solid cancer cells (Fig. 2). In a study conducted in 2012, healthy donors' NK cells were purified, and their exosomes were isolated to assess the cytotoxicity of NK cell-derived exosomes against hematologic cell lines (Jurkat and K562) and also cell lines from solid tumors (SKBR3 and DAUDI). This report indicated that NK cell-derived exosomes induced cytotoxicity in hematologic cell lines, while the cell lines deriving from solid tumors appeared to be relatively resistant to NK cell-derived exosomes' cytotoxicity [217]. Another study investigated the cytotoxic effects of NK cell-derived exosomes on MCF-7, SupB15, CHLA-136, CHLA-255, and NALM-6 cell lines. Their results showed that the percentages of dead cells increased after incubation with NK cell-derived exosomes [218]. In addition to these, the cytotoxic effects of NK92MI-derived exosomes on different leukemic cell lines were confirmed by Samara et al*.* NK cell-derived exosomes also indicated effective therapeutic effects in vivo [223]. After analysis of the tumor-targeted homing ability of NK cell-derived exosomes, high uptake was detected in the tumor site of the hepatocellular carcinoma-bearing mice. In addition, the tumor growth was suppressed by NK cell-derived exosomes. Taken together, the results of different reports exhibit that NK cell-derived exosomes can exert similar effects of NK cells on tumor cells.

Since the mechanism of action of NK cells and their exosomes against tumor and virus-infected cells is relatively similar, it seems that NK cell-derived exosomes use the following mechanisms to fight against virus-infected cells.

NK cell-derived exosome's mechanism of action can be direct or indirect. Indirectly, these exosomes affect other immune cells and activate them to exhibit immune responses against cancer cells or virus-infected cells. Directly, the exosomes elicit cytotoxic effects using the effector molecules that exist in them.

Interestingly, it was shown that NK cell-derived exosomes can affect non-activated NK cells, and these vesicles can activate the NK cells. Shoae-Hassani et al*.* indicated that NK cell-derived exosomes could overexpress the NK activating receptors NKp30, NKp44, NKp46, and NKG2D and be able to educate naïve NK cells, which evolve into a memory-like state, with increased cytotoxicity and enhanced tumor-killing capacity [224]. Dosil et al*.* performed next-generation sequencing for analysis of the small RNA content of resting and in vitro-activated NK cells and the exosomes secreted by them. Then, in silico-predicted mRNA targets were done to identify the immune-related molecules that these small RNAs can target. First, it was clear that NK cell-derived exosomes were rich in miR-10b-5p, miR-155-5p, or miR-92a-3p. Since T cell response can be the putative target of these small RNAs, the CD4^+^ T cells were isolated and treated with NK cell-derived exosomes. The results showed that these exosomes promote Th1 differentiation via Gata3 downmodulation and T-bet de-repression correlating with increased levels of miR-10b-5p and miR-92a-3p. In addition, NK cell-derived exosomes can activate the CD4^+^ T cells and increase IL-2 and IFN-γ production but not Treg responses.

On the other hand, the polarization of monocyte to monocyte-derived dendritic cells can be affected by NK cell exosomes, and these vesicles increase the expression of MHC-II and CD86 on the surface of these cells. So, the antigen presentation and T cell activation can be improved [225]. NK cell-derived exosomes can shift macrophage polarization to M1. It was shown that the expression of iNOS and arginase-1 were increased and decreased, respectively [226]. NK cell-derived exosomes harbor specific cargo that acts on the TGF-β pathway, relieving immunosuppression [227, 228]. In addition, the exosomes secreted by NK cells can suppress the expression of PD-1 and inhibit the exhaustion of T cells in chronic viral infections and tumors [228, 229].

As mentioned, the existence of FasL and TRAIL on the surface of NK cell-derived exosomes and cargo of perforin, granzyme A, granzyme B, and granulysin were confirmed by multiple studies supporting direct cytotoxic effects of NK cell-derived exosomes in the face of target cells.

Killing the target cells can be performed by caspase-dependent or independent pathways. For example, perforin can induce pores in the target cell membrane and contribute to the lysis of the cell or entrance of the granzymes or granulysin into the cytoplasm of the target cell [44]. Granzyme A can damage DNA by cleavage of the SET protein complex or induction of ROS release. Despite granzyme A, granzyme B can activate caspase-dependent apoptosis by direct activating pro-caspase or releasing cytochrome c from mitochondria [228]. In addition, ER-mediated apoptosis can be induced by granulysin [230]. Despite contradictory reports, it seems that using membrane-bound FasL is another mechanism NK cell-derived exosomes use to kill the target cells. A caspase-dependent apoptosis pathway initiates after FasL binds to the Fas on viral-infected or tumor cells. miRNAs are another tool NK cell-derived exosomes use to inhibit target cells [44, 231].

### Effects of exosomes derived from virus-infected cells on NK cells

In addition to immune cells such as NK cells, virus-infected cells can also secrete exosomes that might also affect immune cells. It was shown that these exosomes contain the proteins, nucleic acids, and other components of the original virus. These vesicles can be taken up by other immune or non-immune cells. So they can transfer the virus-related cargo. Some studies reported that exosomes secreted by viruses-infected cells contribute to NK cell-cell dysfunction or activation [232].

Yang et al. isolated exosomes from the serum of chronic hepatitis B (CHB) patients. These exosomes were found to contain HBV DNA, HBV RNA, and HBs Ag. After the isolation and purification of NK cells from the peripheral blood of the healthy donors and CHB patients, they observed that the cytotoxicity of NK cells from CHB patients against K562 cells was decreased compared to the cytotoxicity of healthy donors' NK cells. They also observed reduced CD107a (degranulation molecule) expression on the surface of NK cells. In addition, the secretion of IFN-γ was decreased. So, they concluded that HBV infection suppresses NK cell function. They also assessed whether HBV impaired NK cell function by exosomes. For this purpose, they incubated healthy donor NK cells with exosomes from CHB patients. They observed a decrease in NK cell cytotoxicity, CD107a, IFN-γ, and TNF-α production of NK cells than those incubated with exosomes from healthy donors. This report showed that the expression of the activating and inhibitory receptors on NK cells was decreased and increased, respectively by CHB patients-derived exosomes. Moreover, according to the results of this study, the proliferation of HBV + NK cells was reduced, and they displayed apoptosis [232].

Another study showed that HBV-infected cells release exosomes that contain HBV RNA. These exosomes could upregulate the expression of NKG2D ligands on macrophages by stimulating MyD88, TICAM-1, and MAVS-dependent pathways. Since NKG2D ligands have an essential role in the activation of NK cells, they assessed the function of the hepatic NK cells and indicated that the IFN-γ production of hepatic NK cells in response to HBV only occurs in the presence of hepatic macrophages. So, it concluded that exosomes that are released from HBV-infected cells have an essential role in NK cell activation by increasing the NKG2D ligand expression via macrophages [233].

## NK cell-based therapies in viral infection

**Fig. 3:**
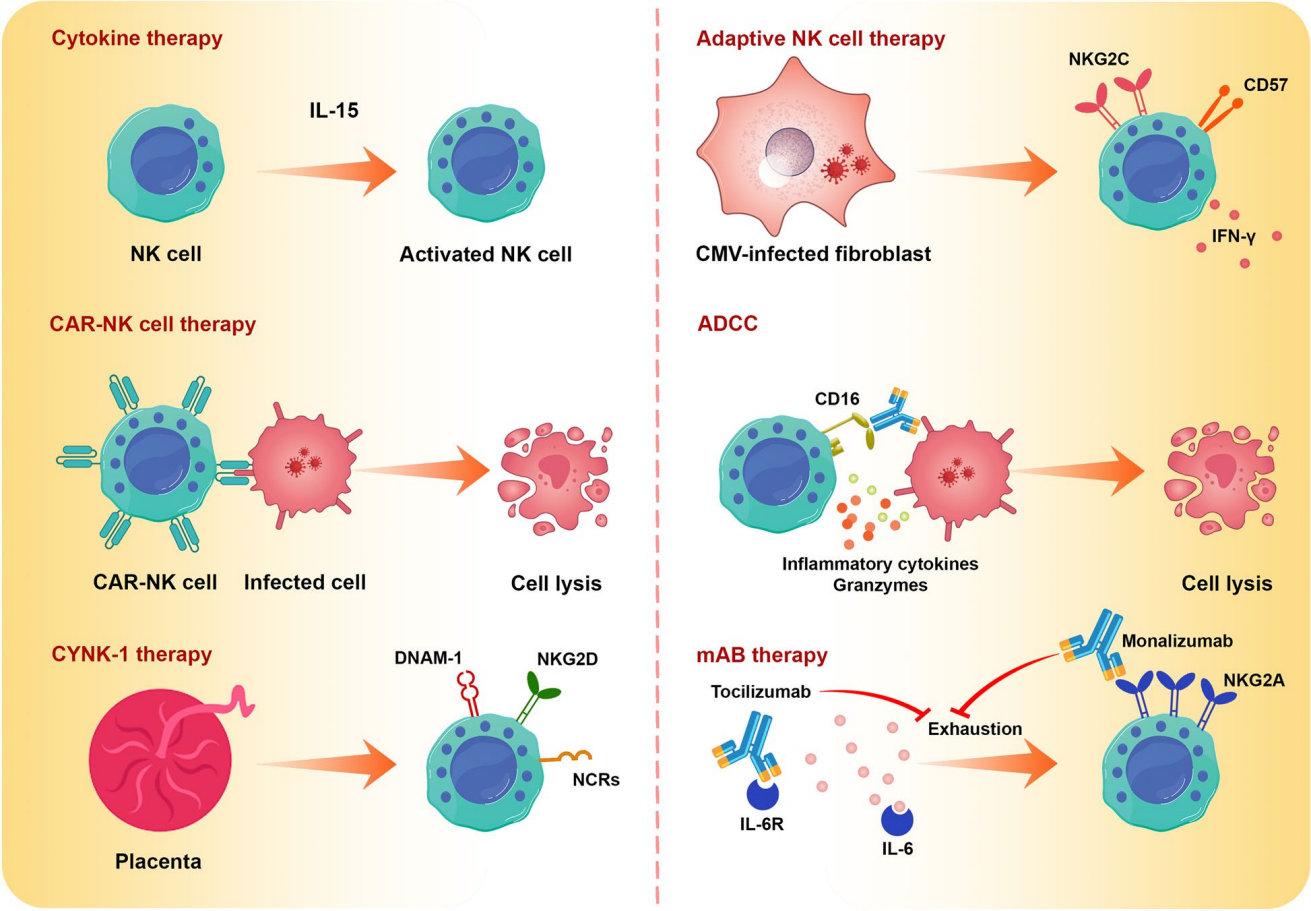
Strengthening the host immune system by therapeutic approaches that augment NK cells’ cytotoxicity and longevity or transferring of the functional NK cells

NK cells have significant roles in the immune response against viral infections that can cause patient morbidity and mortality. Therefore, strengthening the host immune system by therapeutic approaches that augment NK cells’ cytotoxicity and longevity, transferring NK cells, and using NK cell-derived exosomes might be beneficial strategies for infected patients (Fig. 3). Here, we discuss the recent developments in these therapeutic approaches.

### Enhancing the proliferation, cytotoxicity, and lifespan of NK cells

#### Antibody as an NK cell-engager agent

NK cells are the main immune cells that mediate ADCC because the FcγRIII or CD16 is highly expressed on these cells [234]. The use of antibodies against specific antigens is one of the effective approaches in immunotherapy because these antibodies can exploit various arms of the immune system, for example by injection of the antibodies, neutralization, opsonophagocytosis, and ADCC could be occurred that contribute to the clearance of pathogen [234].

The use of broadly neutralizing antibodies against HIV is the potential immunotherapy approach. It was shown that in addition to neutralization, these antibodies could engage activating Fc receptors and enhance protective activity [235]. Passive vaccination by a broadly neutralizing antibody (3BNC117) in mice demonstrated that these antibodies could bind to Fc receptors and induce ADCC; substantially, the clearance of the infected cells was improved [236]. In addition to passive vaccination, active vaccination can induce ADCC-mediating antibodies. Seasonal influenza vaccination of older adults induces ADCC-mediation antibodies that contribute to the augmented ADCC activity against H5 and H7 strains [237]. Another study evaluated the serum samples of people who received a recombinant H7 hemagglutinin vaccine, and serological assays were conducted. It was demonstrated that antibodies induced by H7 vaccination have functional activity in ADCC [238].

NK cell therapy has some limitations; for example, unlike T cells and B cells, NK cells do not have specificity, and the proliferation and viability of these cells are limited in vivo. To overcome these limitations, some interesting immunotherapy approaches were suggested, such as bispecific (BiKEs) and trispecific (TriKEs) killer cell engagers [239]. BiKEs that bind to CD16A on NK cells and HIV-1 envelope glycoprotein-expressing cells could kill the infected cells. Li et al*.* generated a BiKE that consists of CD16A-binding antibody domains fused with one-domain soluble human CD4. Using this BiKE against HIV-infected cells could activate NK cells and induce cytokine production and degranulation [240].

#### Preventing the NK cell exhaustion

It was shown that in chronic viral infections or various cancers, the NK cells became exhausted, and the function of these cells was impaired. The exhausted NK cells upregulated the expression of inhibitory receptors and immune checkpoints. It was shown that classical NK cells’ receptors like NKG2A, KIRs, and LIRs were increased in chronic viral infections or cancers. In addition to this, recently, it was demonstrated that the NK cells could express various immune checkpoints such as CTLA-4, PD-1, B7-H3, LAG-3, TIGIT, CD96, TIM-3, Siglec-7/9, CD200, and CD47. Since the immune checkpoint blockade (ICB) indicated significant results in the activation of T cells and control of cancers and infectious diseases, the use of ICB for enhancing the function of NK cells was investigated, and it was shown that the ICBs could augment the cytotoxic function of the NK cells [241]. The antibody known as Monalizumab, which hinders NKG2A, has been shown to have positive impacts in the treatment of various forms of cancer. As NKG2A plays an essential role in the exhaustion of NK cells, it seems that monalizumab can be considered as NK cell ICB [242]. Blocking the NKG2A enhanced the activity of human NK cells and eliminated HBV infection in mice [71]. Additionally, IL-6 plays a crucial role in the exhaustion and dysfunction of NK cells in individuals with COVID-19. Consequently, the utilization of Tocilizumab, an IL-6 inhibitor, can help prevent the exhaustion of NK cells [243, 244].

#### Cytokines as NK cell activating agents

Activation of the endogenous NK cells by cytokines is one of the challenging approaches in viral infections and cancers. Many cytokines were used for this aim, such as IL-2, IL-12, IL-15, and IFN-α [245]. IL-12 and IL-15 secretion by monocytes was decreased in COVID-19. So, it was shown that the administration of these cytokines can compensate for the NK cell dysfunction resulting from reduced cytokine secretion [246]. Garrido et al. investigated the effects of IL-15 treatment on NK cell stimulation and effector function. ADCC, IFN-γ production, and anti-viral activity of NK cells were improved [247].

### Transfer of functional NK cells

Transferring the functional NK cells is one of the promising therapeutic approaches in viral infections. Different sources of NK cells, such as autologous, haploidentical, adoptive, and off-the-shelf cryopreserved NK cell products, were used for this aim. The clinical trials in this field are summarized in Table 2.

**Table 2:** A summary of NK cell-based clinical trials

Row	Study Title	Trial No	Interventions	Locations	Status
1	Ph1 Study of FT538 Alone and With Vorinostat for Persistent Low-Level HIV Viremia	NCT05700630	Biological: FT538 Drug: Vorinostat	USA	Not yet recruiting
2	Allogeneic Natural Killer (NK) Cell Therapy in Subjects Hospitalized for COVID-19	NCT04900454	Biological: DVX201	USA	Recruiting
3	Off-the-shelf NK Cells (KDS-1000) as Immunotherapy for COVID-19	NCT04797975	Biological: KDS-1000 Other: Placebo	Not yet been determined	Withdrawn
4	Fase I Clinical Trial on NK Cells for COVID-19	NCT04634370	Biological: NK Cells infusion	Brazil	Unknown status
5	Safety Infusion of NatuRal KillEr celLs or MEmory T Cells as Adoptive Therapy in COVID-19 pnEumonia or Lymphopenia	NCT04578210	Biological: T memory cells and NK cells	Spain	Unknown status
6	Natural Killer Cell (CYNK-001) Infusions in Adults With COVID-19	NCT04365101	Biological: CYNK-001	USA	Active, not recruiting
7	Study of FT516 for the Treatment of COVID-19 in Hospitalized Patients With Hypoxia	NCT04363346	Drug: FT516	USA	Completed
8	A Phase I/II Study of Universal Off-the-shelf NKG2D-ACE2 CAR-NK Cells for Therapy of COVID-19	NCT04324996	Biological: NK cells,IL15-NK cells,NKG2D CAR-NK cells,ACE2 CAR-NK cells,NKG2D-ACE2 CAR-NK cells	China	Unknown status
9	CMV Infection and Immune Intervention After Transplantation	NCT04320303	Biological: expanded NK cells	China	Recruiting
10	NK Cells Treatment for COVID-19	NCT04280224	Biological: NK Cells	China	Recruiting
11	Long-term, Non-interventional, Observational Study Following Treatment With Fate Therapeutics FT500 Cellular Immunotherapy	NCT04106167	Genetic: Allogeneic NK cell	USA	Recruiting
12	Adoptive Transfer of Haploidentical NK Cells and N-803	NCT03899480	Biological: Haploidentical NK Cells	USA	Completed
13	Adoptive Transfer of Haploidentical Natural Killer Cells and IL-2	NCT03346499	Biological: NK cells and IL-2	USA	Completed
14	Immunotherapy of Natural Killer (NK) Cells in Human T Lymphotropic Virus Type 1(HTLV-1) Associated Myelopathy(HAM)	NCT02961712	Biological: NK cells Biological: amniotic epithelial cells	China	Unknown status

#### Adoptive NK cells

Recently, some evidence has somewhat changed the views regarding the NK cell as one of the components of innate immunity. The first indication referred to the studies that evaluated the CD94^+^NKG2C^+^ NK cells in HCMV-infected patients and showed that adaptive-like and memory-like NK cells could respond to CMV infection in mouse models and humans [248]. Indeed, adaptive NK cell refers to a group of NK cells with memory properties such as higher longevity, cytotoxicity, and cytokine secretion. The expression of NKG2C, CD57, and IFN-γ is higher in these cells than in normal NK cells [249]. In a study, the severity of COVID-19 in kidney transplant recipients was compared in recipients infected with SARS-CoV-2 and HCMV, or SARS-CoV-2 alone. It was shown that the severity of COVID-19 is higher in the second group. This report can correlate with the effects of adaptive NK cells on COVID-19 [250]. Adaptive NK cells can be prepared in the laboratory using HCMV-infected fibroblasts. So, these cells might be considered a new weapon for virus eradication or infected cell elimination [251].

#### Allogenic and haploidentical NK cells

Autologous NK cells or haploidentical healthy donors’ NK cells can be considered for transferring the NK cells in virus-infected patients. One of the major limitations of this aim is the low count of NK cells in peripheral blood [239]. Additionally, the cytotoxicity of these NK cells is low. So, strategies should be applied to overcome this issue. Oyer et al. designed a particle-based method for expanding cytotoxic NK cells from peripheral blood mononuclear cells. These particles are prepared from plasma membranes of K562-mb21-41BBL cells, expressing 41BBL and membrane-bound interleukin-21 (PM21 particles). PM21 could expand NK cells ex vivo and in vivo [252]. Using G-CSF along with optimized cell culture conditions is another suggested method for expanding cytotoxic NK cells [245].

In addition to peripheral blood, NK cells can be obtained from induced pluripotent stem cells, umbilical cord stem cells, and bone marrow [239]. CYNK-001 is a group of NK cells derived from umbilical cord stem cells. These cells have anti-tumor and anti-viral properties. CYNK-001 is a laboratory-produced NK cell that recognizes and eliminates virus-infected cells using NKG2D, DNAM, and NCRs [253, 254]. Clinical trials using CYNK-001 for patients with COVID-19 are currently ongoing (NCT04365101).

#### Chimeric antigen receptor NK cells

Chimeric antigen receptor (CAR) is a genetically-designed receptor consisting of antigen binding domain, mostly single chain variable fragment (ScFv), a transmembrane region, and a signal transduction domain that is different in each generation of the CARs. The immune cells, mostly T and NK cells, can be genetically modified by CAR transduction and express this receptor on their surface. One of the most significant advantages of CAR NK cells over normal NK cells is their ability for specific recognition of the target cells [255]. In a study, the hematopoietic progenitor cells were transduced with CAR and then differentiated into functional NK and T cells. These differentiated cells prevented HIV infection in humanized mice. It was shown that CAR-NK cells could detect and eliminate mimetic HIV-infected cell lines [256]. Recently, a clinical trial has been underway to treat COVID-19 patients using CAR NK cells that express the angiotensin-converting enzyme 2 (ACE2) receptor on their surface (NCT04324996). Moreover, a modified CAR NK cell was developed to enhance its survival by secreting IL-15 and targeting the S protein of SARS-CoV-2 through the expression of a CAR with an extracellular domain of ACE2. This modified cell has exhibited promising effects against VSV-SARS-CoV-2 chimeric viral particles and the recombinant SARS-CoV-2 spike protein subunit S1. It enhances cytotoxicity and promotes the production of TNF-α and IFN-γ [257].

### Therapeutic potential of NK cell-derived exosomes in viral infection

Using exosomes as a treatment option is becoming favorable among scientists. A study by Sengupta et al. aimed to assess the safety and efficacy of utilizing exosomes derived from bone marrow mesenchymal stem cells (MSCs) as a potential treatment for COVID-19-infected patients. Following four days of treatment, significant improvements were observed in both laboratory test results and clinical symptoms, along with elevated lymphocyte count, with no adverse effects reported [258]. Another study showed that Milk exosomes can bind to monocyte-derived dendritic cells (MDDCs) through DC-SIGN (dendritic cell-specific intercellular adhesion molecule-3 grabbing non-integrin), thereby inhibiting HIV-1 infection in MDDCs and preventing the subsequent transfer of the virus to CD4 T cells [259]. Additionally, exosomes released by virus-infected cells may be used to trigger NK cells. For instance, Hepatocytes infected with HBV release exosomes containing HBV nucleic acids. These exosomes can trigger the activation of NK cells by stimulating specific pathways like MyD88, TICAM-1, and MAVS, resulting in the expression of NKG2D ligands on the cell surface [233].

As we mentioned, NK cells can be used as treatments for viral infection; however, the research on the role of NK cell-derived exosome in treating viral infections are rare, and more studies are needed to shed light on this issue. Because these exosomes can exert similar effects of NK cells and have favorable properties for defense against viral infections.

## Conclusion

In conclusion, NK cells are essential members of antiviral immunity. Their ability to recognize and destroy infected cells using various mechanisms, including releasing cytotoxic granules and producing cytokines, is essential in fighting against viruses. Recently, studies have delineated that the role of exosomes in the antiviral response of NK cells is essential since they can modulate signaling pathways involved in NK activity. Also, some ncRNAs can enhance NK cells' cytotoxic activity and cytokine production. In addition, NK cell-secreted exosomes can inhibit viral replication and boost the antiviral response of other immune cells, such as T cells.

Therapeutic approaches targeting NK cells are attractive options and have shown promising outcomes in treating viral infections. Immunotherapies relying on NK cells, including the transfer of ex vivo expanded NK cells and using allogenic and haploidentical NK cells, have shown promising results in clinical trials. However, further research is required to investigate the potential side effects of these approaches and optimize them to achieve a higher yield. Also, a comprehensive understanding of interactions between ncRNAs, exosomes, and other factors in regulating NK cells in antiviral immunity seems necessary. Despite these challenges, targeting NK cells remains an engaging approach to alleviating viral infections, especially when currently available treatments have limited efficacy or adverse effects.

## Perspective

Activating and inhibitory receptor interactions on NK cells and the function of cytokines and other signaling molecules in controlling NK cell function will be the main topics of future research on NK cells and viral infection. It may be possible to create targeted therapies by identifying particular exosomal cargoes and non-coding RNAs involved in controlling NK cell function during viral infection. Promising research areas include the creation of NK cell-based therapies specifically tailored for each patient and the application of cytokines and exosomes as targeted treatments for viral infections.

## Data Availability

Not applicable.
